# Change detection in pictorial and solid scenes: The role of depth of field

**DOI:** 10.1371/journal.pone.0188432

**Published:** 2017-11-27

**Authors:** Tingting Zhang, Harold Nefs, Ingrid Heynderickx

**Affiliations:** 1 Changzhou Key Laboratory of Robotics and Intelligent Technology, College of Internet of Things Engineering, Hohai University, Hohai, China; 2 Interactive Intelligence Group, Department of Intelligent Systems, Delft University of Technology, Delft, the Netherlands; 3 Institute of Educational Sciences Child and Family Studies, Faculty of Social Sciences, Leiden University, Leiden, the Netherlands; 4 Human Technology Interaction Group, Department of IE&IS, Eindhoven University of Technology, Eindhoven, the Netherlands; University of Akron, UNITED STATES

## Abstract

This paper investigates the influence of depth of field on change detection in both pictorial and solid scenes. In this work, a within-subjects experiment is conducted using a flicker paradigm, with which the hit rate and response time for change detection are obtained. The results show that depth of field has effects on change detection: the hit rate is smaller and response time is longer in the scene with small depth of field than in the scene with large depth of field or uniform blur. It is concluded that when depth of field is small and binocular disparity is not zero in a picture, the influence of depth of field on change detection is more significant than binocular disparity. This conclusion leads to the result that the change in the sharp area is detected easier and faster than in the area that is closer to the observer.

## Introduction

Literatures have revealed a surprising inability for human to detect substantial changes under natural viewing conditions. This phenomenon is now known as ‘change blindness’ [1–4]. Change blindness suggests that limited information in a retinal image is stored as an enduring form across a saccade. Here saccades represent rapid and ballistic movements of the eyes when the point of fixation changes. Saccades can be elicited voluntarily, but occur reflexively whenever the eyes are open, even when fixeated on a target [5]. Change blindness is against the traditional view that a complete representation of a real-world scene is constructed by our visual system [6]. Investigating change blindness and change detection can contribute to a better characterization of the nature of the visual representation, visual attention, and visual memory of the real world.

### Present study

The earliest research on change blindness was on discovering the existence of change blindness [3,7–9], and then researchers focused on creating the experimental methods [1,4,10]. Later, researchers investigated the extent that humans were blind to large changes in contexts that simulate real-world perception [11–13]. More details of the history of the research on change blindness could be found in Rensink’s paper [14]. In these studies, it was found that change blindness could be introduced by many techniques such as gap-contingent techniques [1,7,10,13], saccade-contingent approaches [15–17], shift-contingent techniques [18], blink-contingent techniques [19], splat-contingent techniques [20,21] and so on. Researchers also found that many factors could facilitate or inhibit change blindness and change detection, including the fixation position [6], scene context [22], location of the change [23], and interestingness of the region [1]. One of the important findings in the literature was that changes in the foreground (closer to the observer) could be detected easier and faster than changes in the background [23]. The position of a change is mainly cued by depth cues; however, there are always multiple depth cues in real situations, leading to a question of whether these depth cues play the same or conflicting roles in change detection. We do not find any work directly focusing on investigating the effects of pictorial depth cues on change detection.

Depth of field, as a pictorial depth cue, is defined as the distance range in which objects are perceived as sharp [24–26]. Manipulating depth of field is a popular photographical technique that is used widely by photographers and cinematographers to direct viewers’ attention to interesting objects or important parts of a scene [27–30]. Researchers concluded that the sharp area in a scene with limited depth of field could attract viewers’ attention. For example, Khan et al. [27] used a head mounted eye-tracker to show that observers looked at objects in focus and neglected the blurred background. Su et al. [31] reduced the spatial variation of texture using power maps, high-order features describing local frequency content in an image to reduce the salience of distracting regions. Their work showed that attention could be directed to the area in a picture with high-frequency components. Urban et al.[32] investigated the importance of spatial scale to visual attention through an eye-tracking experiment and concluded that intermediate spatial frequencies (0.7–1.3 cycles per degree) attracted attention, with some variability in the frequency range between different content categories (e.g., whether the photograph depicts a street or open countryside). In addition, researchers concluded directly that the items and regions with a greater level of detail had the observers' attentional priority [33–35].

### Rationale

Previous studies indicate that the fixation positions in a scene with a small depth of field are mainly in the sharp area under free-viewing conditions. In a scene with sharp foreground and blurred background, a change occurring in the foreground can be detected faster than in the background. There are probably two reasons. The first reason could be that the sharp area is more informative compared to the blurred areas. The observation may lead to the hypothesis that changes in sharp areas can be detected easier and faster than in blurred areas and change blindness is experienced more often in blurred areas because sharp areas hold attention [36,37]. The second reason could be that the foreground is closer to the observer [25, 37]. However, there could be conflicting situations. In most pictures, the focal plane is chosen such that the foreground is sharp and the background is blurred with a limited depth of field; however, this is not necessarily always the case. For pictures where the focal plane is not in the foreground, the foreground—closer to the observer—is blurred, while the background—farther away from the observer—is sharp. The question then is which of the two findings related to change detection prevails. Are changes more easily detected in the background since it is sharp (according to the findings of Baveye et al.[29]) or are changes more easily detected in the foreground since it is closer to the viewer (according to the observations of Jansen et al. [38], or Mazza et al. [23])?

In a scene with a large depth of field, the sharp area is large and the effect of depth of field on depth perception is weak. Other monocular depth cues such as relative size, texture, or perspective can also distinguish the foreground from the background, and so, result in changes closer to the observer to be detected faster [23,38]. In a scene that is uniformly blurred, the objects are less informative than in a scene with a large depth of field. The hypothesis could be that changes in a uniformly blurred scene are more difficult and slower to be detected than in a scene with a large depth of field.

Apart from the monocular depth cues, binocular depth cues may make the allocation of attention more efficient and then facilitate change detection [39,40]. The impact of binocular disparity on visual attention has already been extensively demonstrated [41–44]. For example, Itti and Koch[42] suggested that stereo disparity could be a relevant attentional cue. Other studies were even more specific and suggested that the visual system focused attention to a particular depth cued by binocular disparity [43,45]. However, these studies are still insufficient to answer the question whether the combination of binocular disparity with depth of field affects change detection similarly to only binocular disparity or only a limited depth of field. The main purpose of the current work is to reveal the processing priority of various depth regions in complex scenes that contain multiple pictorial cues such as depth of field and binocular disparity.

### Current work and predictions

In the current work, we conducted a within-subjects experiment to investigate how depth of field and binocular disparity influence change detection. The ‘flicker paradigm’ was used to produce change blindness in our work [1]. In this paradigm, two similar images are presented in an alternating sequence separated by an intervening interval. In the flicker paradigm, the change between the two images can be addition, deletion, displacement or other alteration. We only used object appearance as the change. Hence we did not need to consider whether the type of changes would introduce confounding effects. Using this paradigm, we were able to measure the change detection rate and record the response time.

To achieve the purpose of this work, we first created stimuli with different depth of field to investigate the effect of depth of field on change detection. Since depth of field directed a viewer’s attention to sharper areas in a scene [27,46] and change detection required attention, we hypothesized that for a small depth of field change detection was facilitated in sharp areas, while suppressed in blurred areas. As such, it could also be inferred that the chance to experience change blindness was higher in pictures with a small depth of field than in pictures with a large depth of field. Apart from having a difference in attention, the difference in sharpness or detail visibility between in-focus and out-of-focus areas in the picture may have an impact on change detection. We could make the hypothesis that change detection was more difficult and taken longer time in blurred pictures than in sharp pictures. Hence, it was also necessary to compare the effect of a limited depth of field to the effect of overall blur in the picture in terms of change detection. We named the first main factor as depth of field, which included three levels-small depth of field, large depth of field, uniform blur. The second main factor was called position. The position of the change was either in the area that is close to the focal plane or in the area that was far away from the focal plane. The focal plane was always in the middle of the scene (in terms of depth), and so, the area that was far away from the focal plane could be either in the foreground or background, as such also manipulating the distance in depth to the viewer. In addition, the third main factor was named as viewing condition to investigate the effect of binocular disparity on change detection. We performed our investigation with both pictorial scenes and solid scenes (used in the remainder of the paper to refer to non-stereo scenes and stereo scenes respectively).

## Method

Delft University of Technology Ethic Committee approved the research. All participants gave written informed consent prior to the participation.

### Participants

Seventeen male and eighteen female participants took part in this experiment. Their age ranged between 21 and 35 years with a mean of 26.8 years and a standard deviation of 3.3 years. All participants had normal or corrected-to-normal vision, and none of them had color vision deficiencies. All participants gave written informed consent prior to the participation. This study was in line with ethical regulations of Delft University of Technology, Dutch Law and the Declaration of Helsinki.

### Apparatus

Stimuli were presented on a 39'' 3D LG television screen. Stereo and non-stereo stimuli were viewed in the same experimental set-up. For the stereo pictures, the 3D TV was set to its “Side by Side” 3D mode, and the participants wore passive, circularly polarized glasses. For non-stereo stimuli, the TV was on normal 2D mode and the participants viewed the stimuli directly.

### Stimuli

**Fig 1:**
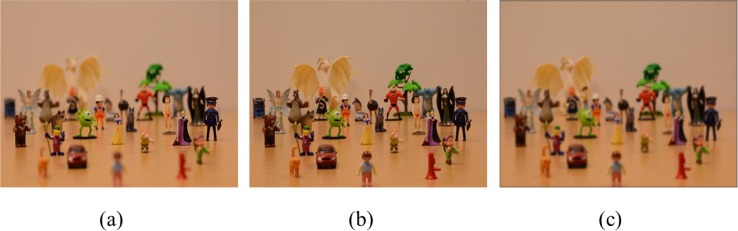
Examples of the stimuli created with different depth of field. (a) Small depth of fieldwith the aperture size of F3.5, (b) large depth of field with the aperture size of F16, (c) uniform blur.

We took pictures of a scene consisting of a group of 28/29 scattered figurines as shown in Fig 1. The pictures were taken with a full-frame Nikon D7000 camera in combination with a Nikon AF–D 50mm lens. We selected an aperture setting of F3.6 to create small depth of field (i.e., more blur) and of F16 to create large depth of field (i.e., having the whole picture relatively sharp), which are shown in Fig 1(A) and 1(B) respectively. Fig 1(C) shows an example of a picture with uniform blur. These pictures were created by convolving the picture having an aperture of F16 with a Gaussian blur kernel. The width of the Gaussian kernel was determined by the maximum blur in Fig 1(A) (i.e., by calculating the blur circle on the object that was farthest away from the focal plane). As a result, the Gaussian blur kernel had a radius (i.e., at 1 standard deviation) of 4.68 pixels. This kernel was subsequently used in all the scenes for creating uniform blur over the whole picture.

**Fig 2:**
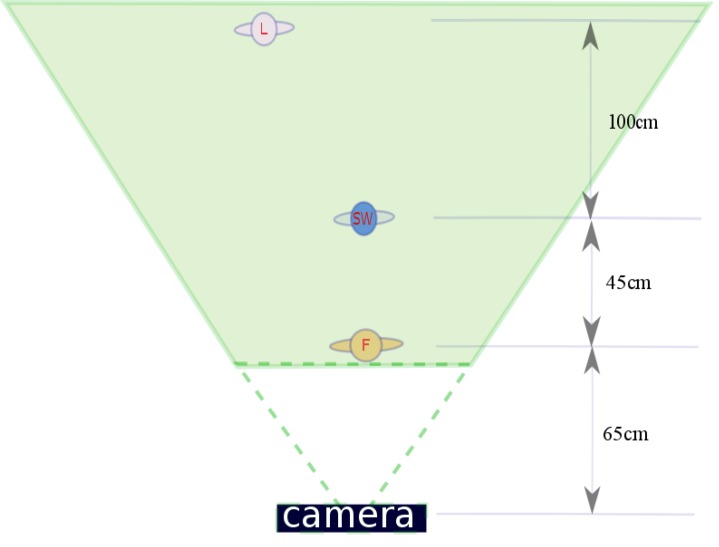
A diagram of the setup of the scene. The object named “SW” shows the position where the camera focused on, the object named “F” is the closest to the camera and the object named “L” is the farthest away from the camera.

When taking the pictures, the “Snow White” figurine that positioned at the center of the scene was at a focal distance of 110cm from the center of the camera lens. The “Snow White” was selected as the center randomly. In the scene, the frontal depth (that is, the depth from the first object to Snow White) was 45cm, and the posterior depth (that is, the depth from Snow White to the farthest object) was 100cm.The setup used to make the pictures is shown in more detail in Fig 2.

Non-stereo and stereo pictures were created separately. For the latter, the focal plane was again set at the position of the Snow White figurine. Left and right stereo half-images were taken with a stereo base of 2.1 cm, in line with the viewing distance and magnification on the display screen in the experiment. That is, pictures were shown with the same visual angle to the participants, as the original scene would have if the observer were at the location of the camera. The cameras were set to converge on the object that was the closest to the camera. The screen disparity for the closest object was hence zero degrees. As a consequence, the closest object was situated at the distance of the display screen and all other objects in the scene were thus situated to be behind the display screen. Although the stereo pictures were toed-in, the actual distortions were small. Earlier tests revealed that this kind of pictures did not lead to visual discomfort [47].

**Fig 3:**
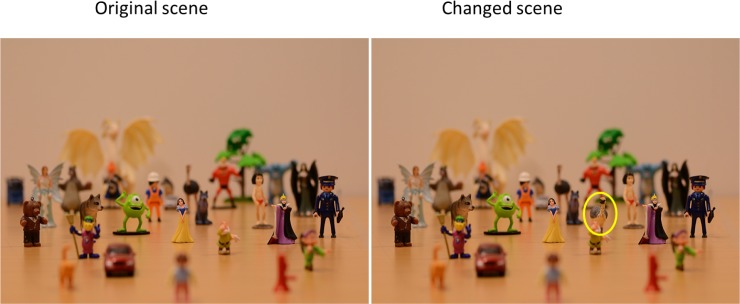
An example of an original scene and a changed scene. The object in the right picture in the yellow circle is new and regarded as the change compared to the original scene.

**Fig 4:**
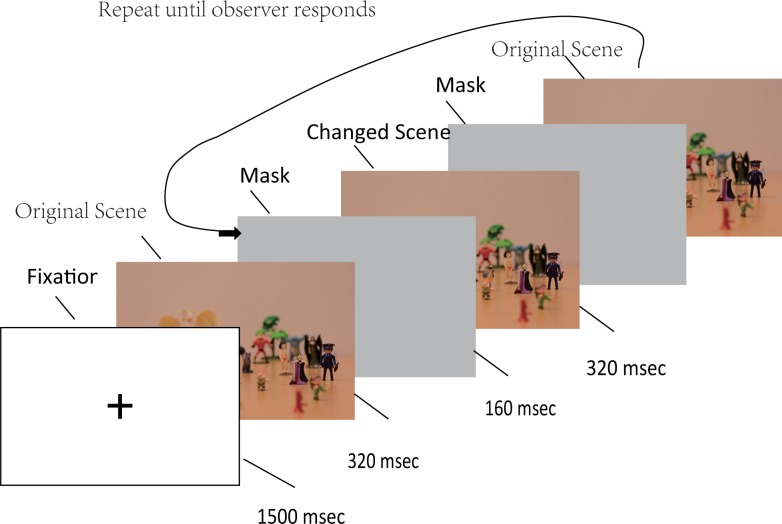
A sample trial for this flicker experiment. After an initial fixation, a sequence of pictures is shown, alternating between an original scene and a changed scene (displayed for 320 msec each), separated by a grey mask (displayed for 160 msec).

The actual trials consisted of a sequence of pictures, based on the same scene, but either with or without a change in the alternating pictures. In other words, the stimuli were generated from two slightly different pictures, as shown in Fig 3. The first picture contained the original scene and was presented for 320 milliseconds, followed by a grey mask for 160 msec. Then the second picture was shown for 320 milliseconds, followed again by the grey mask for 160 msec. In the change trials, the second picture showed a similar scene as the first picture, but with a new figurine added to it (see Fig 3). We call this figurine “changed object” in the remainder of this paper. In the no-change trials, the second picture was exactly the same as the first picture. The sequence of pictures and gray masks was repeated until observers responded. The whole procedure is shown in Fig 4.

**Fig 5:**
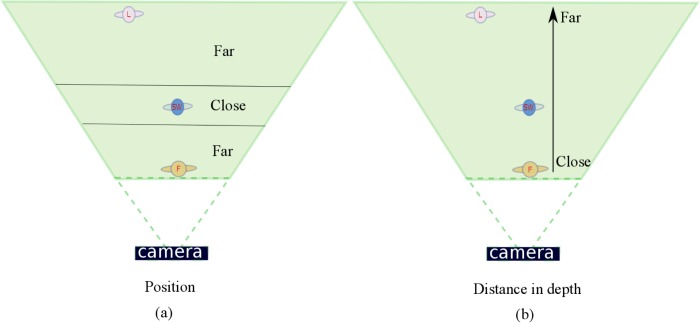
Clarification of terminology used in the experiment. (a) The position of the change can be close to the Snow White (focal plane) or far away; (b) the actual distance in depth of the change may be close to or far away from the observer.

In the experiment, the changed object and its position in the scene were not the same for each condition. We only made sure that for each condition six positions that were close to the focal plane (i.e., with a distance in depth within 10 cm from the plane of the Snow White figurine in the real scene, as shown in Fig 5(A)) were selected and six positions far away from the focal plane (i.e., with a distance in depth beyond 10 cm from the plane with the Snow White figurine) were selected. In both cases, the actual position could be in front of or behind Snow White, as such varying the distance in depth between the changed object and the observer. This latter variation is illustrated in Fig 5(B) for further clarification.

### Procedure

The participant was seated at a distance of 195cm in front of the television screen in a dimly lit room with the illuminance of 16.6 lux, measured horizontally at the desk in front of the observer (with VOLTCRAFT MS-1300 Luxmeter, having a basic accuracy ±5%). No chin rest was used, hence participants were free to move and to choose a position that was comfortable to view the screen and operate the keyboard. The experiment was split into two sessions: one for the stereo trials and the other for the non-stereo trials. Participants could take a 15-minute break between sessions. Seventeen participants first assessed the non-stereo trials and eighteen participants started with the stereo trials.

This experiment consisted of 84 trials in total, of which 42 were stereo trials and 42 were non-stereo trials. Each set of the 42 trials included 36 trials with a change and 6 trials without any change. The no-change trials were included to provide a measure of false alarms, and also to minimize the chance that participants just pressed the button to speed up their responses.

Before the principal experiment, we asked participants to read the instructions carefully and to complete the consent form. After that, there was a short training session, containing four trials, which could help participants become familiar with the experimental interface and task. Two of these four trials contained a change, whereas the other two did not. None of the training trials appeared in the principal experiment.

During the principal experiment, participants were requested to look at a fixation cross at the beginning of each trial (shown in Fig 4) until the first picture appeared. The fixation cross was at the location of the Snow White figurine, and served as a reference to make the initial start location identical across participants. Participants were asked to press the space bar as soon as they detected the change in the trial. It was emphasized that the participants’ only concern was to detect the change as soon as possible. They did not need to spend extra time in identifying and remembering the changed object nor the location. If a participant was confident that there was no change, he/she was asked to press the space bar as well. However, we have to point out that we did not inform participants about being as accurate as possible which may influence participants’ criterion for detecting changes consciously or unconsciously. After pressing the space bar, participants needed to answer the question “Did you see any change in the flickering images?”. If they pressed the “No” key, they would be directed to the next trial. If they pressed the “Yes” key on the keyboard, they had to first answer the question: “which object appeared in the scene as the change?”. Four different figurines, of which only one was correct, were displayed below the question. After this question, they had to answer the question: “In which area did the change happen?”. The area of the scene was separated into four subareas, and participants were asked to pick the correct one. Though participants had to answer the questions about the location and the object of the change, they were told in advance that the answers did not influence the evaluation of their performance. The instruction “Please press the ENTER button on the keyboard to start next trial” appeared on the screen after answering the questions. Participants could take a break between trials as they wanted, and the time to answer the questions was not restricted.

## Results

### Data preparation

To prepare our data for further analysis, we first checked the correctness of the responses. According to previous studies [48,49], no change could be detected below 200ms from the initial presentation of a change. Therefore, any response within 680ms (i.e., 320ms of the original scene plus 160ms of the blank screen plus 200ms) was considered incorrect. Such responses could be misoperations. Based on this criterion, two data points were excluded. Additionally, we did not include one of the participants’ data because 67% of her responses were invalid. The actual reason was unclear, but we considered it likely that this participant did not understand the task well enough.

We did not find any false alarm in the experiment, indicating that none of the participants detected any change when there was no change in the trials. Hence, the rate of false alarms was 0 and the rate of correct rejections was 1, and so these two types of responses were not further considered in the paper. We were mainly interested in the rate of correct hits. The response time for the correct hits was measured starting from the first appearance of the original scene to the time participants responded by pressing the “Space” key. As mentioned in the previous section, participants had to answer two questions in regards to “what & where” after they pressed the “Yes” key. If both answers were incorrect, the response was not considered as a correct hit even if they pressed the “Yes” key. This situation only happened once in our data, and this single data point was considered thus as a miss and was not included when calculating the average response time.

As to the “what & where” questions, we did not discuss the results in details since no special pattern was found. In the stereo experiment, for example, there were 1078 times that participants detected changes which meant that participants answered “what & where” questions 1078 times in total. There were 41 errors in “what” questions and 224 errors in “where” questions. The error rate of “what & where” questions were 3.25% and 17.8%, respectively. The error rate was caculated by dividing the number of errors by 1260 (the number of all the trials with changes). There was no doubt that the error rate of “what” question was very low. The error rate of “where” question was higher, however, this was because some changes happening near the boundary. Therefore, there was no need to discuss the results of “what & where” questions in depth since no special pattern was found.

### Change detection accuracy

**Fig 6:**
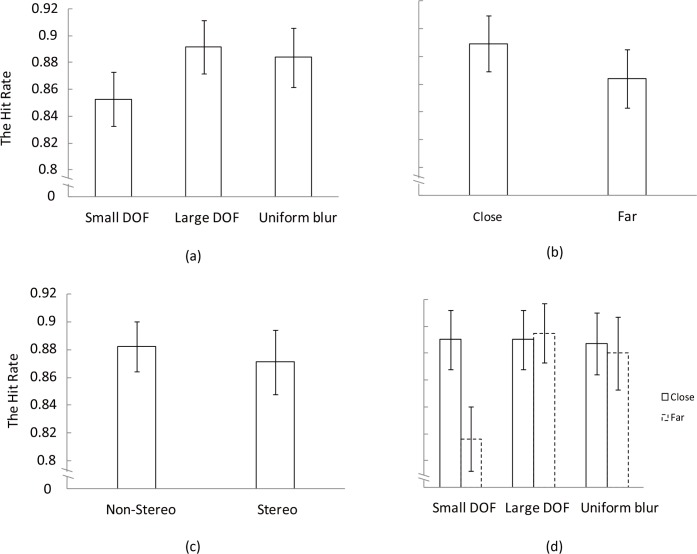
Change detection accuracy in the experiment. (a) Hit rate for different levels of depth of field, (b) hit rate for different levels of position, (c) hit rate for non-stereo and stereo trials, (d) the interaction between depth of field and position. All graphs show the mean ±1 standard error.

We calculated the hit rate (H) as the proportion of correct responses over all responses per participant and per condition. Fig 6(A), 6(B) and 6(C) show the hit rate as a function of depth of field, position, and viewing condition, respectively. Fig 6(A) shows that the hit rate is smaller in pictures with small depth of field than in pictures with large depth of field or uniformly blurred. Fig 6(B) shows the trend that the hit rate is higher in the area close to the focal plane than in the area farther away. It is shown in Fig 6(C) that there is only a small difference in hit rate between non-stereo and stereo pictures. Fig 6(D) illustrates the interaction between depth of field and position, showing that the difference in hit rate for objects close by or far away from the focal plane is only found in pictures with small depth of field, while pictures with large depth of field or uniformly blurred do not exhibit a difference in hit rate depending on the position of the changed object.

The hit rates were entered into a three-way repeated-measures ANOVA, with the factors being depth of field (three levels: small, large and uniformly blurred), Position (two levels: close and far), and viewing condition (two levels: non-stereo and stereo). The main effect of depth of field on hit rate was significant (F (2, 66) = 4.04, p = .022). Pair-wise comparisons between the different levels of depth of field showed that the hit rate was significantly smaller for pictures with small depth of field than for pictures with large depth of field or uniformly blurred. There was no significant difference in hit rate between the latter two sets of pictures. The main effect of position did not reach conventional statistical significance (F (1, 33) = 3.81, p = .059). However, significant interaction between depth of field and position was found (F (2, 66) = 4.78, p = .012). viewing condition was not found to have a significant main effect on the hit rate during change detection.

### Response time

**Fig 7:**
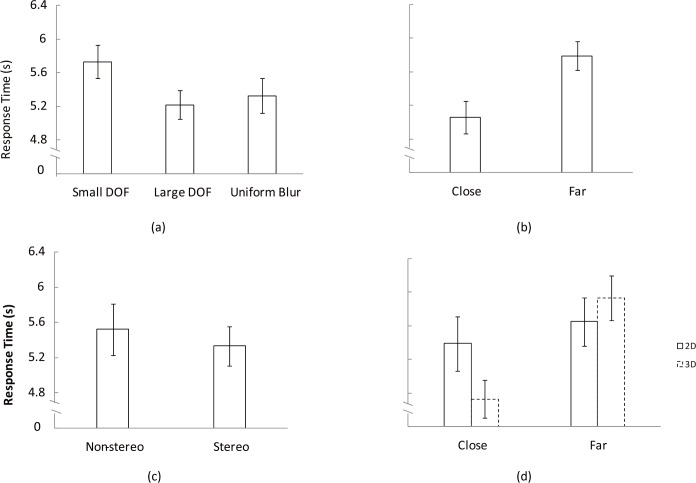
Average response time in the experiment. (a) Average response time across three levels of depth of field, (b) average response time cross positions, (c) average response time for non-stereo and stereo viewing conditions, (d) the interaction between viewing condition and position. All graphs show the mean ±1 standard error.

The effect of the independent variables on the response time is summarized in Fig 7. Fig 7(A) shows that the response time is longer in pictures with small depth of field than in pictures with large depth of field or uniformly blurred. Fig 7(B) shows that a change happening in an area close to the focal plane can be detected faster than in an area far away from the focal plane. Fig 7(C) illustrates that there is a trend that the response time under stereo viewing is shorter than under non-stereo viewing. Fig 7(D) shows the interaction between viewing condition and position, illustrating that the difference between the two viewing conditions is larger in the area close to the focal plane than in the area farther away from the focal plane.

We performed a repeated-measures ANOVA with three factors: viewing condition (non-stereo and stereo), depth of field (small, large and uniform blur), and position (close and far). We found that depth of field (F (2, 66) = 5.46, p = 0.006) and position (F (1, 33) = 24.1, p< .001) significantly influenced the response time for change detection. Pair-wise comparisons between the different levels of depth of field showed that the time to detect a change in the pictures with small depth of field was significantly longer than in the pictures with large depth of field or uniformly blurred. There was no significant difference between non-stereo and stereo viewing conditions. However, the interaction between viewing condition and position was significant (F (1, 33) = 10.51, p = 0.003).

The patterns for hit rate as shown in Fig 6 are different from the patterns for response time as shown in Fig 7, which indicates that speed-accuracy tradeoffs are ruled out in this experiment. Otherwise, short response time (high speed) would lead to low hit rate.

### The dominant factor in change detection: Depth of field or binocular disparity?

**Fig 8:**
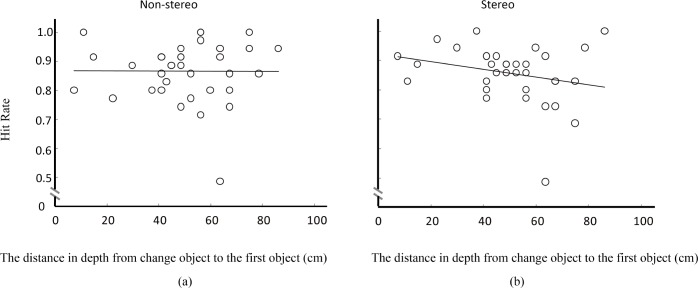
Hit rate as a function of the absolute depth from the changed object to the first object (the object closest to the camera). (a) Non-stereo viewing condition, (b) stereo viewing condition.

**Fig 9:**
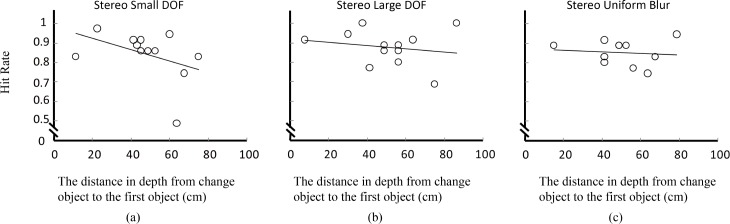
Hit rate as a function of the absolute depth from the changed object to the first object (the object closest to the camera) across depth of field under stereo conditions. (a) Small depth of field, (b) large depth of field, (c) uniform blur.

The effects of position, depth of field, and viewing condition on change detection have been clearly demonstrated, however, the results still could not answer the question that whehter depth of field or binocular disparity is more important for change detection. To answer this question, we analyzed how the actual depth of the change—or the distance of the change with respect to the observer (see Fig 5(B))—influenced change detection. Fig 8 shows a scatter plot of the hit rate as a function of depth from the change to the first object (or equivalently, to the observer if we add a constant distance of 65cm to each data point, as can be deduced from Fig 2). Fig 8(A) shows that there is almost no change on hit rate when the depth of the changed objects increases, whereas Fig 8(B) shows that hit rate decreases when depth increases. Fig 9(A), 9(B) and 9(C) show that under stereo viewing condition hit rate decreases faster with the increase of depth in pictures with small depth of field than in pictures with large depth of field or uniform blur.

**Fig 10:**
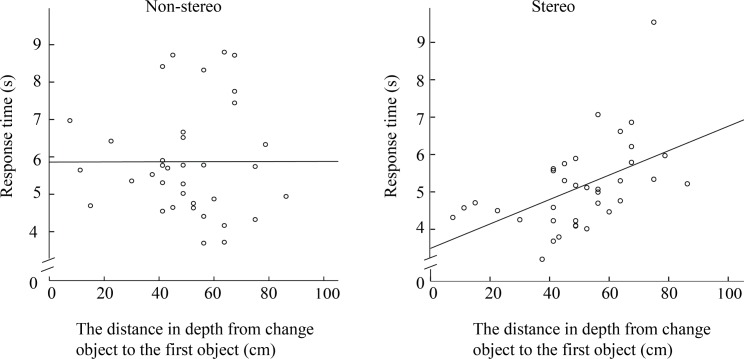
Response time as a function of the absolute depth from the changed object to the first object (the object closest to the camera). (a) Non-stereo viewing condition, (b) stereo viewing condition.

Fig 10 shows a scatter plot of the response time as a function of depth from the change to the first object. Each point represents the mean response time across participants for detecting a change at a specific depth distance. Fig 10(A) shows that there is no obvious linear relationship between the response time and the depth in non-stereo viewing condition, whereas 10(B) shows that the response time increases with depth in the stereo viewing condition. A correlation analysis shows that there is indeed a positive significant relationship between depth and response time under stereo viewing with a Pearson Correlation coefficient of 0.552. Hence, we found that change detection was significantly influenced by solid depth (in stereo viewing), but not by pictorial depth (in non-stereo viewing).

**Fig 11:**
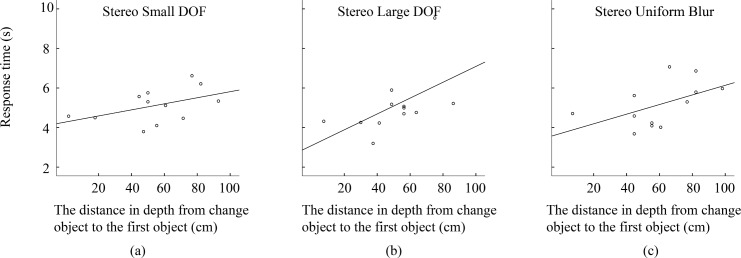
Response time as a function of the absolute depth from the changed object to the first object (the object closest to the camera) across depth of field. (a) Small depth of field, (b) large depth of field, (c) uniform blur.

Furthermore, we analyzed the relationship between response time and the distance in depth of the changed object across depth of field under stereo viewing condition. The results are shown in Fig 11. The correlation analysis shows that there is no significant correlation when depth of field is small. However, response time and the distance in depth of the changed object are significantly correlated when depth of field is large (Pearson Correlation coefficient is 0.677) and also in uniformly blurred pictures (Pearson Correlation coefficient is 0.583). These results suggest that when a picture with a small depth of field is observed under stereo viewing condition, the change detection is mainly influenced by the effect of depth of field but not by the binocular disparity. However, when the effect of depth of field gets smaller (depth of field turns larger), the influence of binocular disparity turns more obvious.

## Discussion

The first principal point of this experiment is that small depth of field has a significant influence on change detection: the hit rate is lower and the response time for change detection is longer compared with large depth of field. There could be different reasons that the hit rate is lower in pictures with small depth of field. First, the lower hit rate could be due to changes occurring farther away from the focal plane are ignored because viewers’ attention is held in the sharp areas. The hit rate for changes close to the focal plane is very comparable to that found in pictures with large depth of field or uniformly blurred, with the hit rate being independent of the position of the change in both latter cases. In other words, there is a higher chance that observers would ignore the change in the area far away from the focal plane when small depth of field generates a considerable blur gradient across the picture. Rensink et al. [1] suggests that change detection occurs only when focused attention is given to the change part. Hence, we may conclude that observers’ attention is predominantly given and held in the sharp area of the scene with small depth of field, even if the observers have to search the whole picture to finish a certain task. Our results are in line with earlier findings obtained with saliency maps based on eye movements, showing that depth of field may direct viewers’ attention [27,28]. However, the low hit rate could also be caused by the limited visibility of details in the blurred foreground or background. If this were the case, we would then expect the hit rate to be also lower in pictures that are uniformly blurred, especially compared to pictures with large depth of field. Our results showed no significant difference in hit rate or response time between pictures with large depth of field and pictures that were uniformly blurred. Hence, we can conclude that blur itself is not sufficient to facilitate the experience of change blindness or exhibit the change detection in situations similar to our experiment. As to response time, we find that it is significantly longer for pictures with small depth of field than pictures with large depth of field or uniform blur. The reason could be that participants move their fixation from time to time across the scene searching for a change in the pictures with large depth of field or being uniformly blurred. In the pictures with small depth of field, large blur gradients may inhibit such visual processing, leading to longer times to detect a change.

In summary, the results in the current experiment strongly support the hypothesis that the chance to experience change blindness is higher in pictures with a small depth of field than in pictures with a large depth of field. However, the results go against the hypothesis that change detection is more difficult and longer in blurred pictures than in sharp pictures. It suggests that the visibility or the blur does not exhibit the change detection in the circumstance similar to our experimental design. These findings show that depth of field is an effective technique to influence change detection.

The second main finding is that position influences the response time for change detection significantly and the effect on the hit rate is at a marginal significance level. Response time is significantly shorter in the area close to the focal plane than in the area farther away from the focal plane. This result also holds for pictures with large depth of field or uniform blur, in which the area that close to the focal plane is not as obvious to participants as in the pictures with small depth of field. The reason could be that most of the changed objects close to the focal plane means close to the center of the image and the initial fixation position according to the settings in this work. As such, our results may confirm the previous studies showing that a change is more easily detected if it appears close to the fixation location [50,51]. Either center of an image or the initial fixation position can be relevant factors, we cannot differentiate the two factors in this experiment. The effect of position on hit rate is relatively weak compared with the effect of position on response time. This result could be caused by other factors that influence change detection such as valence. In this experiment, there are several big objects at the back of the pictures. The appearance of such big objects might also be able to be detected because these big objects looked threatening [52,53].

Viewing condition is not a main factor influencing change detection; we only find a slight, but not significant trend that the response time is shorter for stereo viewing condition than for non-stereo viewing condition. As such, our results confirm the previous finding that observers require the same time to detect a change in pictorial scenes as in solid scenes when using the flicker paradigm [54]. As mentioned in the Method section, the camera was set to converge on the first object in the scene which meant that the disparity for the first object was zero degree. However, in the real world, the zero degree position would be at the focal plane which would be at the position of Snow White in this experiment. Hence, under stereo viewing condition, binocular disparity may influence the effects of other pictorial depth cues on change detection.

In this study, we do not find a significant interaction between viewing condition and depth of field. Nevertheless, we find a significant interaction on response time between viewing condition and position. The response time for change detection is more than one second shorter in stereo pictures than in non-stereo pictures when the change is close to the focal plane. This can be explained by previous studies showing that the visual system is sensitive to binocular disparity in the early visual processing [55,56]. Furthermore, earlier research also shows that the response time may be speeded when there is a valid cue to the location where a change may happen [51,57]. Hence, cueing the viewer more strongly to the focal plane through a combination of binocular disparity and blur—both known to be valid cues for areas close to the focal plane [58]—may account for the shorter response time. Contrary to changes close to the focal plane, the response time is slightly longer for stereo viewing condition than for non-stereo viewing condition when the change is farther away from the focal plane. This may suggest that binocular disparity does not facilitate change detection in areas far away from the focal plane, and instead seems to inhibit detection of change.

To answer the question that whether depth of field or binocular disparity would dominate the change detection when they exist simultaneously, we plotted Figs 10 and 11. The figures show that the distance in depth of the change does not affect response time for non-stereo viewing condition, but it does affect response time for stereo viewing condition; in the latter case it is found that the response time is shorter when the change occurs closer to the viewer holds when depth of field is large or for uniformly blurred pictures. The reason for the shorter response time at closer distance may be related to the larger binocular disparity at increased depth, where the latter requires more effort from eye movements and introduces more discomfort as a consequence of the conflict between vergence and accommodation [59]. Both aspects may lengthen the time to detect a change at larger depth. However, when depth of field is small under stereo viewing condition, response time does not decrease with decreasing distance anymore. It suggests that a small depth of field dominates the change detection when combined with binocular disparity, which indicates that the sharp area resulting from a small depth of field (relatively farther area) has a higher processing priority than the closer area cued by binocular disparity. This finding indicates that the conclusion drawn by previous researchers that observers detect changes happening closer to the viewer faster than changes happening farther away [23,38] only holds when depth of field is large or when the picture is uniformly blurred. However, the stimuli used in the current work lacked many depth cues that appeared quite often in the natural images such as perspective, texture gradient, and so on. If there were more depth cues but not only binocular disparity, the importance of depth information in change detection might change. Hence, the reason that depth of field is the dominant factor in change detection may be that the depth cues are not strong enough.

It is worthwhile to notice that the type of change used in our experiments is the simplest that is in the appearance of an item. It has been found that the performance of change detection depends on the magnitude of the change [60–62]. For example, an object that appears can attract more attention than one that disappears and one might therefore expect onsets to be processed, be detected faster and more easily than offsets. Hence, our conclusions may not be able to apply generally, and the type of chance may interact with the factors that we investigated in the current work.

## Conclusion

The principal conclusion is that depth cues such as depth of field and binocular disparity can both affect change detection separately, however, the influence changes when the two cues exist simultaneously. This work suggests that a small depth of field attracts viewers’ attention to the sharp region in a scene, since changes in the area close to the focal plane or close to the fixation location are detected faster than changes in the area farther away from the focal plane. Although we did not find a main significant effect of viewing condition, we found that the response time increases with increasing depth of the change only under stereo viewing condition, leading to the conclusion that binocular disparity strengthens the effect of depth of field as a depth cue. In the end, we conclude that when depth of field and binocular disparity are combined in complex scenes, the sharp area resulting from a small depth of field has a higher processing priority than the closer area cued by binocular disparity. Depth of field, therefore, is an important image feature that can control the selection of information in pictures under both non-stereo and stereo viewing conditions.

## Supporting information

S1 File This is the data set for non-stereo session experiments. (XLSX)

S2 File This is the data set for stereo session experiments. (XLSX)
